# Impact of COVID-19 on the Internationalisation of the Spanish Agri-Food Sector

**DOI:** 10.3390/foods11070938

**Published:** 2022-03-24

**Authors:** Javier Ballesteros-Bejarano, Ana Cruz González-Calzadilla, Juan Manuel Ramón-Jerónimo, Raquel Flórez-López

**Affiliations:** Department of Financial Economics and Accounting, University Pablo of Olavide of Seville, Ctra. Utrera, Km. 1, 41013 Sevilla, Spain; javier1ballesteros@outlook.es (J.B.-B.); anacgc26@gmail.com (A.C.G.-C.); rflorez@upo.es (R.F.-L.)

**Keywords:** COVID-19, Spanish agri-food sector, exportations, internationalisation, pandemic, lockdown, changes, adaptations, risks

## Abstract

The COVID-19 pandemic has forced companies to suddenly adapt their daily operations. This has also affected the agri-food sector, which is one of the main sectors of the Spanish economy in terms of exportations. Hence, the purpose of this study is to explore how the COVID-19 pandemic has impacted the internationalisation of Spanish agri-food companies. A case study has been developed to gain understanding and insights about the COVID-19 impacts on this sector. The information has been obtained through 12 semi-structured phone interviews that have been made with international/export managers (or managers with international responsibilities) from Spanish exporting companies of the agri-food sector. This research suggests that internationalisation is a great growth opportunity for Spanish agri-food companies. However, COVID-19 has considerably impacted the sector, and they have needed to focus on identifying such impacts to manage them efficiently. In this sense, most interviewees have identified common changes and adaptations to be faced and risks generated by the pandemic that importantly influenced such companies, being the most important risks the sanitary and the payment default risk. In addition, interviewed managers have also explained main actions that were taken. By handling the COVID-19 situation in a flexible, quick and efficient way, companies will achieve international success.

## 1. Introduction

The Spanish agri-food sector has been experiencing a constant international growth during the pandemic, not only increasing its turnover from international operations and exportations, but also in terms of the number of countries reached [1]. This sector has a traditional importance for Spain, which has led it to be one of the most mature sectors in the Spanish economy. The current maturity of the sector and the presence of companies with long tradition which are well-established in the industry are the main reasons explaining the international growth of this sector. In addition, current globalisation, and the need to be as competitive as possible in our capitalist system, or in many cases just to be able to survive, induce companies to look for international opportunities [2,3,4].

Nevertheless, internationalisation has never been easy for companies, especially when they face new markets which differ from the local market [5]. In addition, the international expansion of companies’ businesses in a serious way requires an important investment that companies can lose if they are not able to understand international markets [6]. When companies trade in international markets, they have to deal with completely different people with whom they have to work, and to establish relationships, in the case of clients, and whose preferences and tastes are also different, in the case of consumers. Moreover, logistic chains are longer, where more problems can arise, and there is a wide variety of legislation and government obstacles that do not always make operations easy, affected by political issues as the close of borders and new documentation checks becoming required [7,8,9,10].

Therefore, international markets have always involved a lot of differences with respect to local markets to which companies have needed to adapt in order to keep themselves competitive in the market. Moreover, in this context, the COVID-19 pandemic arrived to cause even more changes and to make more instable and difficult the internationalisation of companies. The virus has increased the number of adaptations that companies from the agri-food sector must carry out to successfully operate in international markets.

Previous authors have also studied the effects of COVID-19 on the agri-food sector, but focusing on specific aspects, such as the supply chain, perishable products, commodity prices or food security policies [11,12,13,14]. Although some previous authors have also studied in some way the COVID-19 impact on the internationalisation of the agri-food sector, it was not found in previous studies which were focused on analysing it in relation with the Spanish agri-food sectors. The Spanish economy relies on this sector, being one of the main sectors in the Spanish economy which generates stable employment, so it is essential to study the COVID-19 impact on this industry, as it represents one of the roots of the Spanish economy and that is why this study differs from others. Most of previous studies are focused on the agri-food sectors of other countries, while research considering both the Spanish case or other countries mainly analyse specific aspects, variables and factors, focusing on specific risks.

Therefore, the purpose of this study is to explore relevant and common effects of the COVID-19 pandemic for the internationalisation of a set of Spanish agri-food companies with an important level of exportations and international activity. In this way, a qualitative overall point of view of the situation will be provided, based on empirical evidence from relevant companies with respect to the studied topic. Moreover, globalisation and international expansion are opportunities for almost every sector, and is also applicable for the Spanish agri-food sector in order to increase sales, reach economies of scale and gain a highly competitive position in the sector. Nevertheless, international agri-food markets have faced many changes because of the pandemic situation regarding environments, challenges, relevant changes, adaptations, factors, risks, problems, etc. In this context, companies needed to face and manage all the difficulties efficiently in order to continue taking advantage of internationalisation and to be successful [15,16,17,18].

This research proceeds with a theoretical development in which an analysis of the industry and a review of previous related literature are made. Then, it is followed by a description of the methodology applied and the explanation of the findings. Finally, some discussions where findings are compared with studies of previous authors, and final conclusions are outlined.

## 2. Theoretical Framework

### 2.1. Industry Analysis

In the last years, the Spanish agri-food industry is showing a solid position within the Spanish economy. According to Spanish Federation of Food and Drink Industries (2020), the production of the Spanish agri-food industry in 2019 was EUR 119,224 million, which represents 2% of the National GDP (Gross Domestic Product) and implies the generation of EUR 26,425 million of gross value added. It is an industry that employs around 436,700 workers within around 30,730 firms [19].

Regarding exports, as it has been published by Ministry of Agriculture, Fisheries and Food [20] in its last annual report, the industry exports continued its upward trend in 2019 reaching EUR 53,180 million. The agri-food industry, with a balance of EUR 14,215 million in 2019, represents 18.3% of the exports of the whole Spanish economy. In this way, Spain was the fourth export country in terms of these kinds of products in 2019, with an export share of 9%, after Netherlands, Germany, and France [20].

Therefore, as it is shown above, exports play a relevant role in the Spanish agri-food industry because of two main reasons. First, around half of the production goes abroad and second, these exports represent a high weight of the overall Spanish exports [20].

According to the data of Ministry of Agriculture, Fisheries and Food [20], the EU-28 was the main destination for the Spanish agri-food exports (72%), and where the exported value reached the figure of EUR 38,108 million, which implied an annual increase of 2.8%.

The first trading partner was France, with 22% of the exporting quota and 26% of importations. Thus, during 2019, products valued at EUR 8391 million were exported to the neighbouring country, which mainly were citrus fruits, pork, fresh vegetables, and olive oil. Within the EU-28, it was followed by Germany with EUR 5885 million (+1.2%). Among the most exported products to that country, the most highlighted ones were citrus, fresh vegetables, red fruits, kiwi and persimmon, and wine and must. Italy stood out as the third destination for Spanish agri-food exports with EUR 5275 million, exporting mainly olive oil, molluscs, pork meat and preserves of fish [1].

Regarding third countries, agri-food exports’ value increased by 12%. The main destinations were China, the United States, Japan, Switzerland, and Morocco. China was the destination of 15.5% of sales of agri-food products to non-EU countries and ousted the United States from the first place, which had traditionally been the first non-EU member trading partner of Spain. Exports to the Asian country stood at EUR 2339 million, with an increase compared to 2018 of 79%, mainly due to the meat of pigs. Exports to the United States reached a figure of EUR 2175 million, which is 7% more than the previous year. US exports are mainly olive oil, wine and must, canned olives and cheese. Japan received merchandise for a value of 1012 million euros (+9.6%), where the exported products were mainly pork, olive oil and wine and must. Finally, Morocco is also an important commercial partner in the agri-food field. It was the fifth destination for exports (EUR 558 million) and the third country in terms of imports (EUR 1544 million). Among the exported products, soybean oil, live plants and live cattle were highlighted [1].

Despite the COVID-19 crisis, the Spanish agri-food industry has remained solid. While the overall data confirm the historical fall of the national GDP in the hardest months of the quarantine, with an historical quarterly decrease of 17.8% and an interannual decrease of 21.5%, the gross added value of the agri-food sector shows a countercyclical growth of 3.6% and 6.3%, respectively. The industry shows its strength and confirms that it gained weight in the second quarter in the economy as a whole, contributing 3.8% of GDP, compared to the contribution of 2.7% registered in 2019 [21].

Regarding the employment adjustment, this sector has not been left out of the generalized crisis, although it was with much less intensity. There has been less job destruction and fewer workers have been affected by an “Record of Temporary Employment Regulation” [19]. In addition, the recovery of the affiliation to the Social Security has been consolidated during the past summer. Taking the second quarter data as a reference again, the number of affiliates in this sector fell by 1.9% annually compared to 4.4% of the total in Spain, while in the agri-food industry the decrease was only 2.4%, while the total decline of the manufacturing industry was 3.7% [19].

The agri-food sector has acquired even more importance during the pandemic, not only with regard to production but also to the entire value chain to be able to supply commercial food establishments and avoid shortage. It has been a challenge that has been overcame by the sector. Nevertheless, the sector continues facing a series of future challenges to respond to needs and demands. Some of the most important future challenges are being able to offer healthy and varied food; contributing to the solution of global obesity; the reduction of food waste; and producing using less products coming from the petroleum [22].

According to Montoriol-Garriga [23], the agri-food sector is the main sector of the Spanish industry. It has strong roots in the territory, what generates stable employment, and it is very open to the outside world. It is also characterized by having a highly atomised business structure, dominated by small companies, and with large companies that are less productive than their European competitors. Montoriol-Garriga [23] also highlights that increasing business size and boosting the productivity of larger companies, through investment in R&D and the adoption of new technologies, would help to increase the competitiveness of a key sector for the economy and society.

The sector, in addition, generates wealth not only through its own activity, but also by indirectly benefiting the rest of the economy thanks to its interrelation with other sectors. These indirect effects are originated through the economic activity from the purchases to its suppliers. To satisfy the demand, suppliers increase purchases from their own suppliers, which also generates added value. The so-called input–output tables of the Spanish economy estimated by the INE are used to compute the indirect effect. In this way, it is obtained that, for every EUR 100 of added value directly originated by the agri-food industry, the economy indirectly generates additional EUR 153, the highest “multiplier” among the industrial branches. Logically, agriculture, livestock, and hunting products are the sector’s main suppliers, followed by wholesale trade services and energy suppliers, but other sectors apparently less linked to the agri-food industry are also relevant, such as legal and accounting services, or chemical ones. In this sense, the direct and indirect contribution of the agri-food industry is equivalent to 7.3% of the total Gross Value Added of the Spanish economy [23].

### 2.2. Literature Review

Previous researchers have developed deep analyses about the importance of internationalisation for agri-food companies [3,24,25,26]. For the companies of this sector, the internationalisation process is not only important to grow, but in many cases, it is also essential to survive. Therefore, in such cases the internationalisation is a need rather than a simple growth opportunity [3]. Preciado et al. argued that one of the roots leading to internationalisation of such companies is the globalized environment that companies are facing nowadays, which makes them to have the need of operating outside [4]. In this sense, internationalisation helps companies to reduce costs through economies of scale, which at the same time, causes companies to become more competitive. Nevertheless, being more competitive is not just a matter of selling more and achieving such economies of scale, but at the same time it is essential to develop best accounting and control practices to make efficient the expansion, namely for smaller companies expanding abroad [27].

Other reasons leading companies to internationalisation are the improvement of their image and the diversification of risks due to the fact of being in different markets at the same time [25,26]. However, even if internationalisation offers many opportunities, it does not offer just opportunities, but also many challenges and factors to be taken into account in a different way from the national market. For instance, Segovia-Villarreal et al. confirmed that when exporting agri-food products, cooperation toward differentiation and positioning is an essential aspect to be considered to internationally succeed, as companies are not as well-known abroad as they are locally [28].

Moreover, it is also important to consider that there are companies which already have an important market share in their own country, being the international process highly motivated because local west agri-food markets are already mature markets, and then, internationalisation is the only way to keep growing [25].

Therefore, previous studies suggest that internationalisation is highly important for agri-food companies, and especially for Spanish ones, as it was shown in the previous industry analysis. However, the COVID-19 pandemic has changed many aspects of this sector. Considering the COVID-19 pandemic effects on Spanish agri-food internationalisation, Alza and Manayay argued that the volume of exports has not been affected by the pandemic [29]. Indeed, there were some companies whose exportations even grew in 2020, in respect to 2019. Further, logistics of foreign trade has been a relatively critical factor that has affected exports, because although the level of warehouses has not been greatly affected by the pandemic, transport has been so, having effects on exportations. In addition, companies faced problems of their own, such as a lack of staff due to infected workers and stoppages in company activity [17]. Another important conclusion of Alza and Manayay was that strategic alliances and customs procedures have been satisfactorily adapted to the context of the pandemic, helping to sustain exports [29]. Moreover, the exportations have grown in many companies of the Spanish agri-food industry as an alternative to the decrease in national sales and operations with companies of the hostelry sector [30].

However, as an opposite finding from previous literature, according to Quevedo-Barros et al., the protection measures taken by governments at the planetary level, as a result of the health crisis caused by the COVID-19 pandemic, has impacted international trade, since it has caused a great impact on the products supply that has led to a fall in the prices of most of the items in a large part of the economies at the global level, among them those of China and the United States [31]. The COVID-19 pandemic has had an impact on the world trade of all nations, however, Quevedo-Barros et al. concluded that, in economic terms, the measures adopted due to the COVID-19 will strongly impact the income of the poorest nations and those whose economic dependence is highly focused on exports [31]. Considering the agri-food industry of countries as the US and Canada, some changes have come to stay. Maintaining flexibility looking for reaching a higher number of different customers and increasing the automatization of production processes in order to avoid problems with infected workers are the two main modifications that companies plan to keep after the pandemic [11].

Further, within the international agri-food context risk perception was seen as highly asymmetric across geographical regions. Companies with longer cash flow coverage periods and higher values of total assets perceived significantly lower risk levels, as cash and assets functioned as a buffer against the impact of COVID-19. In addition, the resilience of agri-food SMEs to the risks presented by the pandemic was reduced because of the absence of a proactive and preventative stance to risk management. Generally, firms operating in both local and international markets perceived lower COVID-19 risks [32]. Nevertheless, it has been found that the main risks highlighted by previous authors was the sanitary risk related to food security [12,18].

Finally, it is notable to highlight the main types of means available for companies to manage their export risks that were found in previous literature. They can be divided into four categories: Acceptance, Transfer, Avoidance, and Mitigation [33]. Risk acceptance means that the exporting company may decide to bear the risk itself. Risk transfer involves moving the risk from the exporting company to the importing company or customers. Risk avoidance occurs when the company avoids situations or operations that imply certain risks that the company does not want to assume, for example exporting to risky countries like Afghanistan, Pakistan, or Iraq. Finally, risk mitigation is a way to prepare the company to minimise the effects of threats and risks faced by a business [33,34,35].

## 3. Materials and Methods

This inquiry presents empirical evidence that has been obtained from 12 semi-structured interviews. Considering the pandemic situation, the interviews were conducted using digital platforms—such as MS Teams—and by telephone. The objective of such interviews was to gain an in-depth understanding of COVID-19 impact on the internationalisation of the Spanish agri-food sector.

### 3.1. Literature Review

An initial literature review of previous studies related to COVID-19 effects over the agri-food sector exportations and internationalisation was carried out. To this end, several databases were used such as Google Scholar, Science Direct, Emerald Insight, Wiley Online Library, Athenea, and other scientific journals. In the literature review many aspects were analysed mainly separately, for a wide variety of specific niches of the agri-food sector and/or specific companies. Thus, considering the relevance of the agri-food sector for the Spanish economy as well as its exportations, that was explained in the industry analysis, this research tries to explore the COVID-19 impact on the internationalisation of this sector as a whole in order to give a general view of the changes and problems that companies need to handle in order to internationally succeed in spite of the pandemic.

### 3.2. Method Selection

The purpose of this research is to gain understanding and insights of the most relevant impacts that COVID-19 has caused on the internationalisation of Spanish agri-food companies. Then, this research can be classified as exploratory due to the fact that it aims to clarify the situation by conducting a preliminary study with an interactive and open-ended nature. Considering the explicative nature of the purpose of this research, a case study is selected as the best method to achieve the desired output. In these cases, a case study methodology has been proven to reveal the most relevant characteristics of the studied reality [36]. In addition, the semi-structured interview was selected as the best available data collection method because it is in line with the explicative nature of this research, and it is suitable to gain a deep understanding of the situation [37]. Indeed, semi-structured phone interviews were chosen in order to gain a deep understanding of the situation by having preestablished questions and introducing new ones that are considered necessary depending on the interviewee. If surveys had been used, gaining understanding, insights and in-depth information about the situation would have been more difficult.

### 3.3. Case Selection

After reviewing the previous literature, the next step was looking for a pool of interviewees for developing a qualitative study to gain understandings of how the theoretical framework is related to the studied reality. Considering the purpose of this research, Spanish agri-food companies with a wide set of characteristics were selected, taking into account data derived from EXTENDA. Due to the exploratory typology of this research, it includes companies with different sizes, products, geographical locations, destination countries, and age, among other characteristics in order to exclude subjectivity. All interviewed companies are producers. Therefore, even if the agri-food sector is the focus of this study, here we are studying companies that produce final products, while final retailers and suppliers of raw material are completely excluded from the purpose of the present inquiry. In addition, considering the international perspective of this study most of the interviewees were international/export managers, except two of them, who were also managers and whose functions also included relevant international responsibilities as they were general managers in charge of the exports area, not having the company a specialized position dedicated for an export manager.

### 3.4. Data Collection

A tentative interview including open questions which provide the opportunity of answering in an explanatory way to interviewees was drafted after doing the literature review. The semi-structured interview was designed to last around an hour, except one of them lasted half an hour because of the time the interviewee had available was limited. Moreover, interviews were recorded through phone recorder and, afterwards, transcribed in order to process the information in a better way. At the beginning of every interview, absolute confidentiality, anonymity, and the treatment of the information just for academic purposes were ensured.

An interview sample is presented in Table 1 to show an overview of the structure and questions of the interview outline.

When conducting a case study, it is highly important to continuously update methodology according to the new information found during data collection. To this end, from the third interview on, the interview outline was constantly updated including, removing, and reformulating questions, or just going more in depth into questions that were providing the most relevant and interesting answers.

The 12 interviews were conducted between November 2020 and June 2021. They were conducted following the common structure but adapting specific questions according to the continuous explanatory answers of the interviewees. Table 2 exhibits a summary of the interviews’ general characteristics regarding the manager position, time duration, size of the company, and products category.

### 3.5. Data Analysis

Once interviews were performed and transcribed, different word documents as drafts were created to resume relevant information of the interviews in order to identify the main aspects highlighted by interviewees. Afterwards, these filtered interviews were analysed and processed by identifying specific internationalisation aspects affected by the virus. Finally, other word documents called “factsheets” were also created for each relevant aspect (e.g., production process, demand, political changes, sanitary risk, logistics…), in which main points and trends were discussed and argued with the different quotes from the respective interviews. In this way information was clearly classified according to the different aspects in which the COVID-19 impact was more relevant.

The first part of the “factsheets” was called “key points”, in which every observed characteristic related to its respective aspect was stated. The second part was the “main trends”, in which key points were further explained and specific singularities and trends were included. Finally, in the last part called “quotes”, all interviewees’ interventions supporting every point or trend were included [38]. This data analysis method was really useful because information was step-by-step processed and classified, providing an overview perception and a close perception of every singularity.

## 4. Findings

Once all interviews were completed, their transcriptions were completed and analysed immediately, and their respective “factsheets” were created. Here, following the logic of the “factsheets”’ creation, findings are presented attending to the most relevant impacts of COVID-19 on the Spanish agri-food sector internationalisation. In addition, as the objective of this study is to gain a general understanding for the whole sector, findings are mainly focused on common impacts for all, or most, interviewed companies. To this end, most relevant quotes, which were in line with the rest of interviewees, were also included. Further, first of all, a preliminary section is included explaining the international context (main current opportunities and challenges faced by the sector in their international activities) perceived by interviewees, in which the COVID-19 pandemic is situated.

### 4.1. International Context: Challenges and Opportunities

The COVID-19 impact on the exporting activity of Spanish agri-food companies has been conditioned by a set of current affairs which is driving the sector to some trends in international markets. Among these current affairs, it is possible to distinguish between challenges and opportunities. In other words, there are some facts or drivers which are currently influencing the agri-food international market [6]. Some of these facts are creating opportunities to companies, while some others are implying some challenges that companies need to face in order to be internationally successful.

At the beginning of each interview, every manager emphasised the importance of international markets and international trading for their companies. Even if there were some companies whose national activity has much more weight compared to their international activity, the international trading was given great importance anyway:

“Obviously, we have a long history in Spain, where the company’s activity is huge, but we already have a ceiling here… well, we found a ceiling that has been leading to higher marginal costs every year. Therefore, commercial operations have been internationally expanded to nearby countries at the beginning, and yes… the purpose of the company is to go out to all exporting markets because they are where growth opportunities can be found”.

This importance was given because companies consider that nowadays the growth opportunity for the Spanish agri-food industry is in international markets. Their industry is in the maturity phase in Spain, which implies that the marginal costs of gaining a bigger market share are very high in comparison with doing so abroad, where the margin for growth is still much higher. Nevertheless, within the European Union, there are other countries which also have a mature agri-food industry, and that is why some managers wanted to be more specific and revealed that within international markets, best opportunities are found in developing markets, where the agri-food industry is still undeveloped and where they expect to continue increasing their international activity:

“We go more to developing markets because there are more opportunities. Agri-food sector is highly mature in developed markets”.

In addition, other companies argued the same but including the fact that they are national companies with a long history as mentioned before, which means that further development and growth are almost impossible for them in the national market, not only due to the high national development of the industry, but also of the company itself. Regarding this issue, the good point is that every company was aware of this situation. There were no companies which did not emphasise the international opportunity for growth. They even mentioned that this issue is properly set in their strategic management plans and in their annual reports, where the growth of their international activity is also duly analysed, showing upward trends in every company from the last years to the present. In this case, it was stated that:

“At the international level, each year we have increased exports and they have gone from representing less than the 10% of the company’s activity, to represent a 30% in 5–6 years”.

In this sense, regarding sales growth, companies are mostly focused on international trading because of the reasons. However, the national market is still very important for them, and although getting more market share in Spain implies much effort and high marginal costs, it is essential for them to be able to at least keep their current market share, as for most of the interviewed companies it still represents most of their turnover. Moreover, there were few companies whose international activity is already higher than their national activity. These companies were relatively younger and smaller than the rest, and they are companies that were created when Spanish agri-food industry was already a mature industry. They were able to catch some share of the national market, but it is very difficult for them to gain more market share from older and well-known companies which are also well-established in the sector. Therefore, the main objective for these companies is even more the expansion to international markets because they do not have a great market share in the Spanish market which they need to maintain.

Moreover, the fact that the best opportunity for growth is abroad, or what is the same, through the internationalisation of the company, has also been in some way proved by the interviewees. The same interviewees who mentioned the highest growth in their respective companies’ sales during the last years, were also who stated the highest increases in the international activities of their companies. Thus, this issue might be understood as empirical evidence of the international growth opportunity that was consistently highlighted by every interviewed manager. Besides, many managers stressed that Spanish agri-food products have a very good image abroad, which makes the path smoother for agri-food Spanish companies to sell their products abroad, mainly due to the fact that other countries have a tasty and quality image of these Spanish products and tend to be receptive to import them. Some interviewees claimed that:

“The brand “Spain” related to agri-food products is always well seen in the international market, it is sold very easily and at relatively high prices”.

However, as agri-food products made in Spain have the mentioned image, it does not happen the same with Spanish brands. It is not the same as a Spanish agri-food product to be a Spanish brand of agri-food products. Here, the difference is that a Spanish agri-food product can be sold under the international retailer brand, so that the retailer is selling a product made in Spain but using its own brand, which implies that final customers are associating the quality of the product to the retailer brand, even if they can see on the package that it is a Spanish product done by the Spanish company “X”. While, if the international retailer is importing and selling Spanish products of a Spanish brand, the final consumer will associate the quality with the Spanish private brand. Summarising, the main difference here is about exporting products under the retailer brand, that is producing for them, or on the other hand, exporting the products under the private brand of the exporting company. Of course, considering differences in quality according to their respective prices. Regarding this, main retailers of each country are the most important clients for interviewed Spanish agri-food companies in international markets, and currently there is an increasing trend among these retailers of asking to produce retailer brands, which is one of the most important challenges that Spanish agri-food companies are facing when exporting. This is affecting companies because their products are not linked with their brands, which limits the recognition of their own brand. In addition, producing retailers’ brands means lower profitability than exporting private brands. Here, interviewees stated that:

“One of the most important challenges in international markets is that the business models that are progressing faster are mainly based on retailers’ brands or distributors’ brands, so that, our private brands have to compete with these brands that are very powerful. And if we produce retailer brands, margins and recognition are lower”.

This challenge was defined by managers as the ability of understanding their brand outside Spain, mainly understanding that they have not the same brand power abroad than in Spain. Furthermore, it is important to mention that adapting private brands to international markets is very challenging. Private brands must compete with retailer brands, which they highlighted to be more difficult because of the lack of image differentiation. It forces exporting companies to be more innovative to differentiate from retailer brands and to negotiate giving much importance to the image of their brand. Nevertheless, regarding competition with retailer brands, there is an offset between the national and the international market. Although in the national market, Spanish private brands are well-known and it is easier for them to differentiate from retailer brands, Spain is one of the countries with the highest market share of retailer brands. While in international markets their brands are not so well-known, as explained before, but the market share of retailer brands is lower than in Spain.

Finally, yet importantly, interviewees also give considerable importance to other challenging adaptations that Spanish agri-food companies have to carry out when exporting. Some of them were controlling costs very well, identifying the best advantage of operating in each country (product preferences, low taxes, possibility of producing in the country…), making exportation an important business and not something complementary for the company, and adapting the products and brands to the importing country.

### 4.2. COVID-19 Impact on Internationalisation of Spanish Agri-Food Sector

As mentioned before, the interviews did not include specific questions related to what is presented below. The following sections were created after realising that the great majority of interviewed managers highlighted almost the same set of international aspect that are relevant with respect to the COVID-19 impact. Here, it is also important to underline that not every manager gave the same importance to all the aspects revealed below. However, all of them were given high importance by the great majority of them. Therefore, following this logic, the main changes and adaptations that companies need to face during the pandemic, as well as the international risks that the COVID-19 has intensified are presented below, which are the main COVID-19 impacts that were found affecting most of the companies and what can be more properly generalised to the rest of the sector.

#### 4.2.1. Main Changes and Adaptations to Be Faced Due to COVID-19

As it has been introduced above, the COVID-19 pandemic required companies to face a set of changes during their internationalisation process. Interviewees mentioned that when their companies carry out exporting activities, it is not the same as operating in the national market but just further away. They are going to different markets, which have completely different characteristics and they need to adapt to every single country where they export. Besides, with such an extraordinary event as the COVID-19 pandemic, the number of changes and adaptations required to go abroad have been even higher. Companies have had to face a set of circumstances that they never faced before.

The COVID-19 crisis has created an uncertain environment completely new for them. To confront the pandemic crisis, companies have had to be very flexible to manage the crisis quickly and efficiently. Interviewees stated that they normally take decisions having in mind the long-term more than the short-term. However, the newness of the situation made them to focus on the immediate future more than in the long-term.

The COVID-19 pandemic has brought a crisis with some characteristics that managers of companies had never seen before. Interviewed companies explained new adaptations of their products, production processes and operations related to new requirements because of the pandemic. For instance, regarding their products, the most common new adaptations have been related to labels and packages, which must be changed because clients required a COVID-19 stamp certifying that the food and its production process are secure from the virus, mainly at the very beginning.

Moreover, all companies had to adapt their production processes and plants to the pandemic crisis. For instance, related to work shifts, many companies highlighted the implementation of higher frequent work shifts because of the need of stops in order to implement cleaning protocols. In addition, other adaptations of working processes were also needed to ensure health safety of workers and sanitary safety of products. In many cases, companies had to handle with infected workers, what implied production stoppages and quickly hiring replacement staff for two or three weeks. These aspects, as well as increasing the number of buses for workers due to seats limitations and in order to avoid mixing workers with third people from public transport, continuous realisation of COVID-19 tests, or equipping employees with proper clothing were important adaptations that companies need to implement to face changes generated by the pandemic, and which implied important increases in costs.

“To sum up, in international markets, we need to adapt 100% to our clients according to their new requirements because of the COVID-19 pandemic, what supposes an important challenge”.

However, these products’ adaptations are not easy to implement. In order to be able to carry out all required adaptations, companies also needed to invest in research and development and in employee training to adapt their production processes as quickly and efficiently as possible. Nevertheless, adapting production processes is inherently a costly effort for companies, although it is not equally complex for every company. According to interviewees, it was easier for smaller firms because their production facilities are normally smaller and more flexible, while it was more complex for large firms, which have big and technologically complex factories that are already optimized to benefit from economies of scale as much as possible. Therefore, these adaptations to the COVID-19 pandemic have increased in general terms the production costs of exported products from large companies because the implementation of such changes was more complex and costly due to their highly specialised factories:

“Well, the initial challenge was that efficiency was lost, especially in large companies like us. There are companies that are smaller and may have had more flexibility. In factories like ours, which are huge, highly technical and with very large volumes, it was a challenge to adapt the production losing the least possible efficiency”.

Moreover, exporting changes and adaptations that companies have had to face when trading abroad during the pandemic were not just related to their products and production processes, but also to political and cultural aspects. Considering political aspects, interviewees underlined that international commerce was slower or even stopped in many cases due to borders restrictions and, in addition, more documentation was needed, what increase the bureaucracy and complexity of exportations. Furthermore, cultural differences were other important international issue that companies need to manage during the pandemic. It was pointed out that each country had different changes with respect to the tastes or ingredients demanded during the pandemic, which had direct relation with the products’ adaptations mentioned before. In addition, cultural differences also affected in how negotiations were conducted, as some countries started to demand more requirements and other less. Further, interviewees stressed that having local teams in destination countries considerably helped companies to get a better understanding of changes and cultural differences and how the pandemic was going on. In this sense, a relevant quote was:

“Regarding culture, there is a part that I have told you before about habits, like we like orange and others like apple, what also implied different changes in demand depending on the country. And there is another cultural part of the negotiation. Negotiating with a Chinese is not the same as negotiating with an Arab or an American. All of them started to demand new different requirements related to the COVID-19. So, you must adapt to preferences and cultures, and that is why I told you before that the main basis of international business are in people with experience and who know the dynamics of negotiation with different cultures in dynamic environments”.

According to interviewees, many people think that agri-food companies increased their profits in 2020 during the lockdown. However, developing adaptations as quick as possible involved increases in costs that were translated into almost not any increase in benefits for agri-food companies which suffered the respective increase in demand. Regarding this, interviewees claimed that:

“In the end, you have a peak of demand, it is true, but it does not benefit you at all because operations cost you much more than before. I mean, moving factories that can also be infected, or stopping the factory workers get infected are very costly issues. You also have to spend more money in cleaning, dressing, employees shifting, you have to put buses because otherwise they mix with people over who you don’t have control, it is a must to provide EPIS, COVID-19 tests… Moving and organizing industrial operations that involve many people in a big factory is very expensive, because it cannot be tele-worked”.

Among all the COVID-19 crisis singularities, a common consequence of every crisis has also taken place. The COVID-19 crisis has caused a change in consumers behaviour and preferences which have affected companies:

“To the extent that this economic crisis will produce changes in consumer buying behaviour and companies, we will have to be attentive to accommodate our offer to the changes that can occur in consumer behaviour, because of a decrease in available income among other factors. In that sense, we can say that it is the most immediate future that we have to face now”.

This change in consumer’s behaviour and preferences was translated in changes in demand that companies have had to face. Unexpected changes in demand usually affect companies negatively if they are not ready for them. This could happen in both situations; sharp demand increases and decreases. The most common well-known negative impact is a decrease in demand, which results in companies decreasing their sales and losing an operating volume that can cause companies to lose the advantages of economies of scale and dilute fixed costs. However, a sharp increase in demand is also a risk for companies because if they are not able to supply such an increase in demand, they can lose the business. During the virus lockdown, both scenarios occurred to companies. Demand for basic need products increased sharply, while some non-basic need products demand decreased a lot. For instance, interviewees pointed out the following about demand:

“Normally your company is adapted to certain levels of demand. Then, when demand changes a lot like in the lockdown, you have to adapt the factories and production processes and sometimes it is not easy”.

“The demand does not usually have such sharp peaks as now in the pandemic, they tend to be more attenuated curves and to which you can adapt over time whether they increase or decrease. But the pandemic is being so extreme, it is very important to forecast and adapt new demand as good as possible”.

As in every crisis period, the available income of householders has been reduced during the COVID-19 pandemic crisis, forcing them to change their preferences and to prioritise basic needs, what also exists among agri-food products. In this sense, as mentioned before, companies producing basic need products received a severe demand increase to which they had to be as responsive as possible, increasing production while adapting their facilities and losing workers for some weeks because of the virus. At the same time, some companies producing non-basic need products suffered a decrease in demand, while others received an increase in such demand due to a shift from hostelry sales to some non-basic need products from supermarkets. It has basically depended on the specific type of non-basic need products. Nevertheless, most companies which suffered a decrease in national demand were able to maintain sales thanks to an increase of international sales in new markets. In addition, all companies highlighted that during the lockdown, they found more opportunities in international markets, regardless the type of product, what led them to increase their international activity and gave more importance to it. Therefore, the internationalisation of agri-food companies has played an essential role for such companies during the pandemic crisis in order to survive and even to grow. Companies look for new international markets demanding their products that were suffering a decrease in demand because of the pandemic. It was a clear way of transforming an important threat into an opportunity.

As it was previously mentioned, non-basic need products suffered more the crisis consequences, while basic need products’ demand increased. This is, in some way, in line with what many interviewees also stated about retailer brands. They asserted that the production of products for international retailer brands has been the part of companies which suffered less effects from the pandemic contingencies, in fact it was even favoured by such demand increase.

Moreover, without considering if companies’ products are basic need products or not, all companies were affected by a decrease in sales, such as the hostelry industry, which was even completely closed. In addition, travel retail was also cancelled, which in this case affected more to companies producing non-basic need products. The decrease, or almost the cancellation of sales to these two niches also contributed to the explanation of why agri-food companies almost did not increase profits during the pandemic lockdown period. Moreover, the reduction of these sales was even more significant for companies to increase their focus on expanding exportations towards new international markets and, in this way, offsetting them.

Thus, it is possible to state that thanks to the increase in international trading that companies looked for, some of them were able to maintain their sales or even keep growing during the pandemic situation:

“During the lockdown we experienced an important decrease of national demand. Then, the company tried to increase international operations, and thanks to an increase in exportations we were able to keep the company’s turnover”.

Finally, other important aspect of internationalisation considerably affected by the COVID-19 pandemic was international logistics. Due to the virus, logistics problems started to arise mainly with China. Containers stopped arriving to China, which caused a huge containers deficit in China. As there were no containers in China, whole ships with empty containers started to be sent to China. Therefore, interviewees stated that operational and logistics costs increased significantly, while times also increased. Moreover, as this situation derived in a containers’ shortage, the price per container also increased until it was duplicated. In this sense, interviewees whose companies exported to China stated the following:

“Since there were no containers in China, they sent ships full of empty containers to China, so if you wanted to get on the ship to carry products the cost was much higher. The operational cost was increased because in the end it is different to share a ship in which the costs are going to be distributed among all the companies that have a space on that ship, than taking the empty container shipping company to China. Then if you want to take one your cost will be much higher, being affected till the point that the price was almost double comparing with few months ago. A few months ago, a freight to China could cost you around 1000 or 1100 euros, in confinement it rose to 1300 and now it goes for about 2100 euros. So that container deficit is another factor that affects us a lot.”

#### 4.2.2. Main Risks Faced during the COVID-19

In the internationalisation process, there are new risks that companies need to face and handle properly if they want to keep alive and succeed. The COVID-19 bring some of such risk beyond the normality. There were some risks that interviewed managers underlined regarding their increasing international importance during the COVID-19 pandemic, and especially during the lockdown. In this line, the risks to which companies interviewed gave such greater importance were two: sanitary risk and payment default risk.

The most highlighted risk that was mentioned as the most relevant one during the pandemic by most of the companies was the payment default risk. Most companies agree that during the pandemic, there were companies whose sales were reduced and started to have liquidity problems. In this context, the payment default risk has increased its frequency. Further, it is important to consider that a payment default is more difficult to claim when it occurs in another country. Normally, payment default is the main source of losses for companies. They are occurring with higher frequency in developing markets, where, at the same time, business opportunities are also higher. However, there are also developed markets, such as the US, where this risk is also high due to the reason that sometimes there are companies that do not fulfil their payments because they know that carrying out the necessary legal processes to claim the default will not be worth to the creditor company as it is so expensive (even more than the amount defaulted). In addition, it was perceived that, in this pandemic situation, companies started to mistrust more new clients regarding payment defaults, demanding sooner payments to them and allowing less amounts for pending payments. Regarding this issue, interviewees claim:

“The main risk during the COVID-19 pandemic is being the payment risk. You have to try to have the risk insured either in an insurance company or with a form of payment that ensures your credit, such as a letter of credit”.

“Regarding payments, it is the risk about which we are more worried always, and during the lockdown it has become even more important. We have started to mistrust more new clients and clients from developing countries, which normally fail more payments. However, in some countries like the US, this risk is being also increased as legal issues are very expensive, there are companies that do not pay you because they know that you will not claim it if the amount is not very high”.

The second risk to which companies gave more importance during the pandemic was the sanitary risk. In fact, this is a specific risk of the agri-food industry. Companies consider it as important because sanitary issues can heavily damage agri-food companies’ image and therefore, also their brands. European sanitary standards are very similar in all European countries, however, in the rest of countries outside the EU, companies have to make greater efforts to adapt their products to their respective sanitary legislations. This implies that if companies fail when doing so, products can be confiscated, which will lead to important losses for the company. Considering this, with the arise of the COVID-19, companies started to be even more worried about sanitary issues. Interviewees seemed to be so fearful about the possibility of having a sanitary scandal related to their companies during the pandemic. Here the relevant issue is that with the presence of COVID-19, people started to be much more sensitive to sanitary issues, which implied a greater importance of this risk. For example, many workers infected or products with some kind of bacteria, because company reputation can be, according to them, completely destroyed. In addition, bad product conservation by the client can produce sanitary problems that affect the brand, without this being an error of the exporting company, but a mistake of their clients which are the intermediaries between the exporting companies and final consumers. Moreover, regarding sanitary issues, interviewees also stressed that unexpected changes in sanitary legislation can considerably affect companies. The required adaptations, if they are possible, can require modifications till making the market unprofitable and eventually, having to leave it. These changes can heavily increase costs. In this line, interviewees claimed that:

“The greatest risk for us, or what scares us the most is a sanitary or health problem, what has been intensified during the pandemic. We take our products and our processes forward, but logically the logistics chain is long, and things can happen in your customers’ stores, and many things can happen that finally affect your product. Further, sometimes the legislation, depending on the countries, is different, what can also generate any type of problem. For example, listeria in meat products is a risk of intoxication, and sometimes the rates of diphtheria that I handle in Spain in any sausage may be higher than those that can handle in other countries. We are always afraid of coming out in some negative news, as in news of that type, that is what scares us the most… For instance, now if many workers of a company get infected, the image of the company can be highly damaged, and sales will also decrease, what will have much worse effects now, because due to the pandemic people is much more worried about sanitary issues. We offer many guarantees, we never run any risk for that to happen, but such problems can always happen”.

Furthermore, interviewees also mentioned the main risk management systems they are applying in order to face such increasing risks. First, it is important to mention that among the types of risks management systems mentioned by previous authors (risk acceptance, risk transfer, risk mitigation and risk avoidance [33,34,35], the main risk management systems used by interviewed companies belong to risk mitigation systems.

In terms of sanitary risks, companies try to be very strict in quality controls and in following COVID-19 protocols. In addition, some companies carry out a set of preventive measures, such as taking production samples in the different production stages, controlling products’ temperatures along all the logistic chain, or continuous cleaning protocols. For example, to control products’ temperature, they use specific devices with the objective of avoiding responsibilities of products that were not correctly conserved by other companies and which arrive to final customers in bad conditions. It is true that this kind of sanitary risks can affect companies regardless of the pandemic. However, regarding that the pandemic is a distinctive and global health issue, any other problem related with sanitary risks can affect companies much more. Now, everyone is more sensitive to health and sanitary scandals, and that is why managing such issues is more important now than ever.

Finally, with respect to the payment default risk, which was pointed out as the most important risk by companies, they carry out several actions in order to mitigate the risk. Among practices performed with more frequency, it is important to underline: having risk protocols to stop crediting clients, visiting the company before starting a relationship in order to better audit it, assuring the risk in an insurance company, untrusting new clients, asking for sooner and higher payments, asking for a part of the payment before sending the product or even before starting producing it in order to at least compensate costs, and using client ratings to allow more or less credit without assuming high risks. In addition, just a few companies highlighted that they have specific contingency plans for risks, where every single risky event is listed and all actions to be taken are planned.

Regarding risk mitigation of such risks, it is possible to highlight the following quotes:

“We have a super exhaustive quality control policy with all our approved suppliers. And for the business with our clients, we have a monthly control review. For example, we also use specific devices to control the temperature of the products during the transport. Besides our current COVID-19 protocols are very exhaustive and we require the same to our suppliers”.

“Before starting a relationship with a new client, we audit their accounts to avoid possible payment defaults. Nevertheless, we have even stopped the credit to very important clients because they were paying with so much delay”.

## 5. Discussion

The objective of this study was to explore the impact of COVID-19 pandemic on the internationalisation of several Spanish companies from the agri-food sector. The research was conducted to get understandings and insights of the main impacts and aspects that Spanish agri-food companies must face when going abroad and operating internationally during the pandemic situation. The main impacts found were a series of changes and adaptations to be faced, and few risks that were intensified when operating in international markets during the pandemic. The findings were obtained from 12 semi-structured interviews with relevant managers of the companies, in most of the cases export managers. In this section, main relevant comparisons with respect to previous authors will be presented.

Findings about the COVID-19 pandemic impact on Spanish agri-food sector internationalisation suggest that all studied companies increased exportations because of the pandemic. In this way, studied companies offset the decrease in hostelry industry and travel retail. On the other hand, even if the reduction of hostelry sales was also found by Cruz [30], he did not give the same importance to travel retail sales, and it was underlined that in some cases exportations even decrease, although increases were also mentioned [29,30].

In addition, findings suggest an increasing flexibility regarding products and productions processes leading to changes and adaptations to face the current situation and which will not be implemented anymore after the pandemic (workers transport, clothing, shifts, tests, certifications, etc.), which was in line with prior national authors [17]. However, while such adaptations were perceived as something temporary, in the case of companies from other countries, the found idea was looking for solutions to the short-term that can be implemented to improve in the long-term, as higher automatization in factories and a wider offer of products [11].

Logistic problems suggested in this study are in line with previous research. Basically, COVID-19 has damaged the logistic chain, increasing times and availability of resources [29,30]. However, some previous authors also underlined that adopted measures have reduced exportations to poorer countries, where the income reduction was greater [31], which was not found in this study.

Furthermore, according to findings, small companies were able to adapt better and sooner to COVID-19 impacts due to their higher flexibility. Nevertheless, while this research highlights this issue in terms of production adaptations due to less technical facilities and lower production volumes, prior authors did so in terms of risks, leading them to manage more efficiently risks related to COVID19 pandemic [32].

Finally, in this research, two risks have been identified to increase their importance and possible negative effects for companies during the pandemic, having companies to control and manage them closely. These risks are the sanitary risk and the payment default risk. In the case of sanitary risk, it was also found in previous literature as one of the main risks highlighted during the pandemic [18]. However, the increasing importance that interviewees gave to payment default risk was not equally found in previous research. Further, regarding their management systems, prior authors analysed the main risk management systems to face risks, which showed risk mitigation being the risk management system most used by international companies [33,34,35]. In the case of interviewed companies, the main risk management systems used to face both risks were also risk mitigation systems, such as hiring insurances, developing contingency plans, rating clients for credits, or taking samples of production.

## 6. Conclusions

As marginal costs of increasing national sales are high due to the huge dimension and maturity of the Spanish agri-food sector, internationalisation becomes the best opportunity for companies to expand their businesses. However, when taking advantage of the international opportunity, companies needed to suddenly adapt to the new COVID-19 situation, which has led companies to be more flexible and focus on the short term rather than thinking in the long-term, which is easier to achieve by smaller companies as they have less technical factories and lower production volumes. Nonetheless, this flexibility involved an important increase in costs that offset any increase in sales, leading to no additional profits and required to design an appropriate risk management system to manage international risks, being the two most important ones, the sanitary and the payment default risk.

Finally, due to the relevance of the studied sector for the Spanish economy, the interesting international opportunity and the important impact of COVID-19 pandemic for its internationalisation, this research gives a gateway to future research which can analyse the key aspects and risks presented in this inquiry in a deeper and quantitative way in order to reach further conclusions of the Spanish agri-food sector internationalisation, as well as analysing how companies readapt to normality after pandemic.

## Figures and Tables

**Table 1:** Interview sample.

Structure of the Interview	Sample of Questions Used
Personal and company information	Previous work experience Working responsibilities What is the present situation of the company? What is the future of the company?
COVID-19 impacts on internationalisation process	What is the percentage of international activities in the company? What have been the main impacts of COVID-19? What are the main changes of selling products to international clients before and after COVID-19? How has been coordinated and managed international activity with the COVID-19 pandemic? How has changed the way final consumer is reached?What has been the impact of different cultures on businesses? How have relationships with international clients changed? Did relationships with other companies (externalisations, collaborations, other suppliers…) suffered from COVID-19? What external factors influencing the company changed due to the COVID-19? How is the negotiation of international contracts?
International risks related to COVID-19	What are the main risks that your company has to face during COVID-19 pandemic in the international markets? What about legal/political/demand/price fluctuations/sanitary/exchange rate/climate/production risks in relations to COVID-19 crisis? How were those risks managed?
	What are the main risks management systems used by the company?

**Table 2:** Interviews, interviewees, and companies’ information.

	Manager Position	Duration of the Interview	Size of the Company	Product Categories
Interview #1	Export Manager	1:02:14	Medium	Oils
Interview #2	Financial and International Manager	1:51:48	Large	Preserved food
Interview #3	Export and Sales Manager	1:01:12	Medium	Oils
Interview #4	Export Manager	0:54:38	Large	Milk and dairy products
Interview #5	Export and Sales Manager	1:05:29	Large	Meet products
Interview #6	Export Manager	0:53:21	Large	Fruit juices
Interview #7	Export Manager	1:00:22	Large	Chocolate products
Interview #8	CEO	1:13:51	Medium	Chocolate products
Interview #9	Export Manager	1:07:49	Medium	Oils
Interview #10	International Manager	0:33:02	Medium	Drinks and chocolates
Interview #11	Export Manager	0:53:04	Large	Poultry meat products, eggs, and milk dairy products
Interview #12	International Manager	1:08:57	Large	Berry Products

## Data Availability

Not applicable.

## References

[1] Las Exportaciones Agroalimentarias, Pesqueras y Forestales Continuaron su Tendencia al Alza en 2019 y Alcanzaron 53.180 Millones de Euros. https://www.mapa.gob.es/es/prensa/ultimas-noticias/las-exportaciones-agroalimentarias-pesqueras-y-forestales-continuaron-su-tendencia-al-alza-en-2019-y-alcanzaron-53.180-millones-de-euros/tcm:30-542609.

[2] Sanz A.V. (2018). El sector agroalimentario español frente al reto de la internacionalización. Alimentaria. Rev. Tecnol. E Hig. Aliment..

[3] Calderon H., Fayos T., Mir J. (2009). Valoración de las políticas de promoción de la internacionalización del sector agroalimentario. La dirección de empresas ante los retos del siglo XXI. Homenaje al profesor Juan José Renau Piqueras. Publ. Univ. Valencia.

[4] Preciado L.B., Caderón L.F.E., Casella J.A.L. (2011). Gestión del riesgo cambiario en una compañía exportadora. Estud. Gerenc..

[5] Peng M.Y.-P. Understanding Small and Middle Enterprises’ Internationalization Process at Food and Restaurant Service Industry. Proceedings of the 2019 16th International Conference on Service Systems and Service Management (ICSSSM).

[6] Serrano R., Olmos M.F., Pinilla V. (2018). Internationalization and performance in agri-food firms. Span. J. Agric. Res..

[7] Magnusson P., Westjohn S.A., Semenov A.V., Randrianasolo A.A., Zdravkovic S. (2013). The Role of Cultural Intelligence in Marketing Adaptation and Export Performance. J. Int. Mark..

[8] Samiee S., Chabowski B.R., Hult G.T.M. (2015). International Relationship Marketing: Intellectual Foundations and Avenues for Further Research. J. Int. Mark..

[9] Tang C.F., Abosedra S. (2019). Logistics performance, exports, and growth: Evidence from Asian economies. Res. Transp. Econ..

[10] Ulloa I.J.F., Rojas C.E.V. (2014). Diagnóstico de la cadena logística de exportación del banano ecuatoriano hacia estados unidos de américa. Saber Cienc. Y Lib..

[11] Weersink A., von Massow M., Bannon N., Ifft J., Maples J., McEwan K., McKendree M.G., Nicholson C., Novakovic A., Rangarajan A. (2020). COVID-19 and the agri-food system in the United States and Canada. Agric. Syst..

[12] Hossain S.T. (2020). Impacts of COVID-19 on the Agri-food Sector: Food Security Policies of Asian Productivity Organization Members. J. Agric. Sci. Sri Lanka.

[13] Coluccia B., Agnusdei G.P., Miglietta P.P., De Leo F. (2021). Effects of COVID-19 on the Italian agri-food supply and value chains. Food Control..

[14] Zhang Y., Diao X., Chen K.Z., Robinson S., Fan S. (2020). Impact of COVID-19 on China’s macroeconomy and agri-food system—An economy-wide multiplier model analysis. China Agric. Econ. Rev..

[15] Poppe K. (2020). COVID-19 will Change the Agri-food System—But how?. EuroChoices.

[16] Coopmans I., Bijttebier J., Marchand F., Mathijs E., Messely L., Rogge E., Sanders A., Wauters E. (2021). COVID-19 impacts on Flemish food supply chains and lessons for agri-food system resilience. Agric. Syst..

[17] Corchuelo Martínez-Azúa B., López-Salazar P., Sama-Berrocal C. (2021). Impact of the COVID-19 Pandemic on Agri-Food Companies in the Region of Extremadura (Spain). Agronomy.

[18] Montanari F., Arayess S., Barbarasa T., Clavarino A., Ferreira I., Mahy A., Stelios Michałowska A., Schröck C., Servé Arthur A., Wesolowska V. (2020). The Response of the EU Agri-Food Chain to the COVID-19 Pandemic: Chronicles from the EU and Selected Member States. Eur. Food Feed L. Rev..

[19] Informe Económico. http://fiab.es/es/archivos/documentos/INFECO_2019.pdf.

[20] Ministerio de Agricultura, Pesca y Alimentación. https://www.mapa.gob.es/es/default.aspx.

[21] El Sector Agroalimentario Español Demuestra su Solidez en Plena Crisis del Coronavirus. https://www.elconfidencial.com/economia/2020-11-03/sector-agroalimentario-espanol-coronavirus-bra_2809711/.

[22] Los Cinco Retos Futuros de la Industria Alimentaria. https://www.eleconomista.es/aragon/noticias/10552985/05/20/Los-cinco-retos-futuros-de-la-industria-alimentaria.html.

[23] La Industria Agroalimentaria Española: Estructura Empresarial y Productividad. https://www.caixabankresearch.com/es/analisis-sectorial/agroalimentario/industria-agroalimentaria-espanola-estructura-empresarial-y.

[24] Calderón García H., Fayos Gardó T., Mir Piqueras J. (2013). La internacionalización de las cooperativas agroalimentarias. Nece-sidad y problemática. Mediterráneo Económico. Colección Estud. Soc..

[25] Juliá J., García G., Meliá E. (2012). La globalización y los modelos de crecimiento de los grupos cooperativos. Las cooperativas agroalimentarias en España y la Unión Europea. Ekon. Rev. Vasca Econ..

[26] Fayos Gardó T., Calderón García H., Mir Piqueras J. (2011). El éxito en la internacionalización de las cooperativas agroalimentarias españolas. Propuesta de un modelo de estudio desde la perspectiva del marketing internacional. CIRIEC-España Rev. Econ. Pública Soc. Y Coop..

[27] Jack L., Florez-Lopez R., Ramon-Jeronimo J.M. (2018). Accounting, performance measurement and fairness in UK fresh produce supply networks. Account. Organ. Soc..

[28] Segovia-Villarreal M., Florez-Lopez R., Ramon-Jeronimo J.M. (2019). Berry Supply Chain Management: An Empirical Approach. Sustainability.

[29] Alza Collantes C.J., Manayay Azabache C.D.P. (2020). Factores Críticos que Afectan las Exportaciones de la Empresa J&L Agroexpor-Taciones SAC Durante la Pandemia de la COVID-19, Trujillo; Repositorio de la Universidad Privada del Norte. https://hdl.handle.net/11537/25161.

[30] Cruz J. (2020). Las empresas cárnicas se vuelcan en la exportación ante la evolución del mercado nacional por la COVID-19. Eurocarne Rev. Int. Sect. Cárnico.

[31] Quevedo-Barros M.R., Vásquez-Lafebre L.M., Quevedo-Vázquez J.O., Pinzón-Prado L.T. (2020). COVID-19 y sus efectos en el comercio internacional. Caso Ecuador. Dominio Cienc..

[32] Abu Hatab A., Lagerkvist C., Esmat A. (2020). Risk perception and determinants in small- and medium-sized agri-food enterprises amidst the COVID-19 pandemic: Evidence from Egypt. Agribusiness.

[33] Lehmann R., Hauser C., Baldegger R. (2013). Managing Export Risks: Export Risk Management Guidelines.

[34] Yüksel S., Dinçer H., Meral Y. (2019). Financial Analysis of International Energy Trade: A Strategic Outlook for EU-15. Energies.

[35] Sokhatska O., Bashynska I., Stepanova T., Malanchuk M., Rybianets S., Sobol O., Stepanova T.V. (2019). Modelling the risks of international trade contracts. Int. J. Eng. Innov. Technol..

[36] Quinlan C., Babin B., Carr J., Griffin M. (2019). Business Research Methods.

[37] Barrett D., Twycross A. (2018). Data collection in qualitative research. Évid. Based Nurs..

[38] Merly C., Chapman A., Mouvet C. (2011). An End-Users Oriented Methodology for Enhancing the Integration of Knowledge on Soil–Water-Sediment Systems in River Basin Management: An Illustration from the AquaTerra Project. Environ. Manag..

